# (2-{[2-(2-Amino­ethyl­amino)­ethyl­imino]­meth­yl}phenolato-κ^4^ *O*,*N*′,*N*′′,*N*′′′)copper(II) perchlorate

**DOI:** 10.1107/S1600536811020095

**Published:** 2011-06-04

**Authors:** Moussa Dieng, Aliou Hamady Barry, Mohamed Gaye, Abdou Salam Sall, Paulo Pérez-Lourido, Laura Valencia-Matarranz

**Affiliations:** a Département de Chimie, Faculté des Sciences et Techniques, Université Cheikh Anta Diop, Dakar, Senegal; b Département de Chimie, Faculté des Sciences, Université de Nouakchott, Nouakchott, Mauritania; c Departamento de Química Inorgánica, Facultade de Química, Universidad de Vigo, 36310 Vigo, Pontevedra, Spain

## Abstract

The asymmetric unit of the title complex, [Cu(C_11_H_16_N_3_O)]ClO_4_, consists of two Cu^II^ ions coordinated by Schiff base ligands and two perchlorate anions. The Schiff base mol­ecules are linked to the Cu^II^ atoms *via* three N atoms and one O atom, resulting in a square-planar geometry. Inter­molecular hydrogen bonds involving the NH groups as donors and O atoms of the perchlorate anions as acceptors are observed.

## Related literature

For related structures, see: Ambrosi *et al.* (2003 ▶); Jiang *et al.* (2009 ▶).
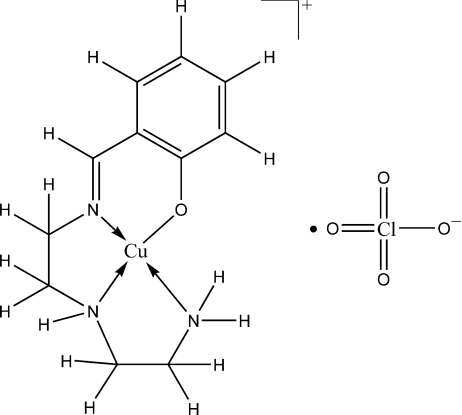


## Experimental

### 

#### Crystal data

[Cu(C_11_H_16_N_3_O)]ClO_4_
*M* *_r_* = 369.26
Triclinic, 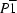

*a* = 10.371 (3) Å
*b* = 12.615 (3) Å
*c* = 13.390 (3) Å
α = 108.240 (4)°
β = 105.568 (4)°
γ = 108.154 (4)°
*V* = 1445.2 (6) Å^3^
*Z* = 4
Mo *K*α radiation
μ = 1.72 mm^−1^
*T* = 293 K
0.14 × 0.12 × 0.10 mm

#### Data collection

Enraf–Nonius CAD-4 diffractometer
9604 measured reflections
4979 independent reflections
4043 reflections with *I* > 2σ(*I*)
*R*_int_ = 0.024
2 standard reflections every 167 reflections  intensity decay: none

#### Refinement

*R*[*F* ^2^ > 2σ(*F* ^2^)] = 0.037
*wR*(*F* ^2^) = 0.131
*S* = 1.04
4979 reflections
403 parameters
6 restraints
H atoms treated by a mixture of independent and constrained refinement
Δρ_max_ = 0.53 e Å^−3^
Δρ_min_ = −0.52 e Å^−3^

### 

Data collection: *CAD-4 EXPRESS* (Enraf–Nonius, 1994 ▶); cell refinement: *CAD-4 EXPRESS*; data reduction: *MolEN* (Fair, 1990 ▶); program(s) used to solve structure: *SHELXS97* (Sheldrick, 2008 ▶); program(s) used to refine structure: *SHELXL97* (Sheldrick, 2008 ▶); molecular graphics: *ORTEP-3 for Windows* (Farrugia, 1997) ▶; software used to prepare material for publication: *SHELXL97*.

## Supplementary Material

Crystal structure: contains datablock(s) I, global. DOI: 10.1107/S1600536811020095/aa2008sup1.cif

Structure factors: contains datablock(s) I. DOI: 10.1107/S1600536811020095/aa2008Isup2.hkl

Additional supplementary materials:  crystallographic information; 3D view; checkCIF report

## Figures and Tables

**Table 1:** Selected bond lengths (Å)

Cu1—O2	1.891 (3)
Cu1—N6	1.933 (4)
Cu1—N5	2.005 (4)
Cu1—N4	2.006 (4)
Cu2—O1	1.895 (3)
Cu2—N3	1.930 (3)
Cu2—N2	2.010 (4)
Cu2—N1	2.012 (3)

**Table 2:** Hydrogen-bond geometry (Å, °)

*D*—H⋯*A*	*D*—H	H⋯*A*	*D*⋯*A*	*D*—H⋯*A*
N1—H1⋯O3*P*^i^	0.85 (2)	2.52 (3)	3.188 (5)	136 (4)
N1—H1⋯O3*P*	0.85 (2)	2.54 (3)	3.208 (5)	136 (4)
N2—H2⋯O9*P*^ii^	0.85 (2)	2.30 (3)	3.069 (6)	151 (4)
N2—H3⋯O2	0.86 (2)	2.12 (2)	2.955 (5)	162 (4)
N4—H4⋯O8*P*	0.86 (2)	2.46 (3)	3.223 (7)	148 (3)
N4—H5⋯O1	0.86 (2)	2.24 (2)	3.084 (5)	168 (4)
N5—H6⋯O6*P*^iii^	0.84 (2)	2.47 (4)	3.081 (5)	131 (4)
N5—H6⋯O4*P*	0.84 (2)	2.51 (3)	3.195 (5)	139 (4)

Symmetry codes: (i) 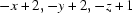; (ii) 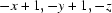; (iii) 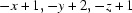.

**Fig. 1.**
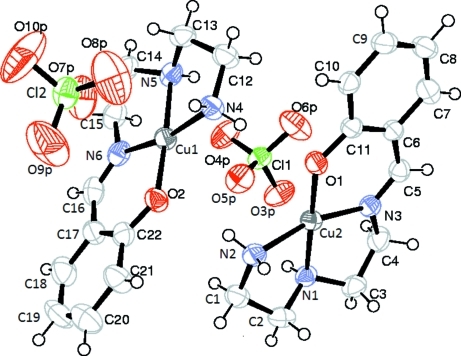
An ORTEP view of the title compound, showing the atom-numbering scheme. Displacement ellipsoids are plotted at the 50% probability level.

**Fig. 2.**
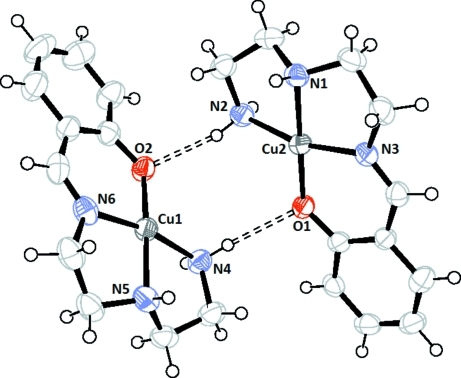
Molecular representation of the compound showing hydrogen bonds. The Perchlorate groups are omitted for clarity.

## supplementary crystallographic information

### Comment

The asymmetric unit of the title complex, [C_11_H_16_N_3_ClO_5_Cu]_2_, consists of two Cu^II^ ions coordinated with Schiff base ligands and two perclorate anions. The Schiff base molecules are linked to the Cu^II^ atoms via three N atoms and one O atom. Each Shiff base ligand exhibits a square-planar geometry about the copper(II) ion.  Intermolecular hydrogen bonds involving the NH groups as donors and O atoms as acceptors are observed. The Cu–O distances are 1.891 (3) and 1.895 (3) A° while the Cu–N are in the range 1.930 (3)- 2.011 (3) A ° . These values are lower than those observed for the copper complex obtained from the ligand 4-chloro-6-hydroxymethyl-2-((3-aminopropylimino)methyl)phenol (Jiang *et al.* , 2009).  The sum of the angles around the Cu1 atom is 359.56° and around Cu2 atom the sum is 360.3°. These facts indicate that there are very slight distortions from the square planar geometry around the Cu^II^ atom. In the two molecules the atoms around Cu are situated in the same plane (dihedral angles N2—Cu1—N1—C8 = 178.4 (3)°, N2—Cu1—N1—C8 =-178.4 (3)° , N5—Cu2—N4—C18 = 178.9 (4)° and O2—Cu2—N4—C19 =-177.4 (3)°  ). The structure of the complex is shown at Fig. 1. Intermolecular hydrogen bonding network is shown at Fig. 2.

### Experimental

Diethylentriamine (1.0311 g, 10 mmol) and salicylaldehyde (2.4408 g, 20 mmol) were dissolved in 20 ml of ethanol with few drops of glacial acetic acid. The mixture was refluxed for 3 h. On cooling a yellow oil was isolated. In a round bottomed flask, copper perclhorate (0.5249, 2 mmol) dissoveld in 10 ml of methanol was introduced. The resulting ligand (0.4145 g, 2 mmol) dissolved in 10 ml of methanol was added. Immediate color change was observed indicating instant formation of the complex. The mixture was stirred at room tempearture for two hours. The blue solution was filtered off and the filtrate was letf at room temperature. After one month, suitable blue crystals for X-ray analysis were obtained. Yield: 70%. Anal. Calc. for [C_11_H_16_N_3_ClO_5_Cu]_2_ (%): C, 35.78; H, 4.37; N, 11.38. Found: C, 35.80; H, 4.35; N, 11.34. Selected IR data (cm^-1^, KBr pellet): 3216, 1637, 1600, 1582, 1197, 764.

### Refinement

The H atoms of the NH and NH_2_ groups were located in the Fourier difference maps and refined with N—H distance restrained to 0.86 (2) A °. Others H atoms (of the CH_2_ groups) were placed geometrically and refined with a riding model. *U*_iso_(H) for H was assigned as 1.2 *U*_eq_ of the parent C atoms.

### Crystal data

**Table:**

[Cu(C_11_H_16_N_3_O)]ClO_4_	*Z* = 4
*M**_r_* = 369.26	*F*(000) = 756
Triclinic, *P*1	*D*_x_ = 1.697 Mg m^−^^3^
Hall symbol: -P 1	Mo *K*α radiation, λ = 0.71073 Å
*a* = 10.371 (3) Å	Cell parameters from 25 reflections
*b* = 12.615 (3) Å	θ = 11–15°
*c* = 13.390 (3) Å	µ = 1.72 mm^−^^1^
α = 108.240 (4)°	*T* = 293 K
β = 105.568 (4)°	Prism, blue
γ = 108.154 (4)°	0.14 × 0.12 × 0.10 mm
*V* = 1445.2 (6) Å^3^	

### Data collection

**Table:**

Enraf–Nonius CAD-4 diffractometer	*R*_int_ = 0.024
Radiation source: fine-focus sealed tube	θ_max_ = 25.1°, θ_min_ = 1.8°
graphite	*h* = −12→12
π scans	*k* = −15→15
9604 measured reflections	*l* = −15→15
4979 independent reflections	2 standard reflections every 167 reflections
4043 reflections with *I* > 2σ(*I*)	intensity decay: none

### Refinement

**Table:**

Refinement on *F*^2^	Primary atom site location: structure-invariant direct methods
Least-squares matrix: full	Secondary atom site location: difference Fourier map
*R*[*F*^2^ > 2σ(*F*^2^)] = 0.037	Hydrogen site location: inferred from neighbouring sites
*wR*(*F*^2^) = 0.131	H atoms treated by a mixture of independent and constrained refinement
*S* = 1.04	*w* = 1/[σ^2^(*F*_o_^2^) + (0.0736*P*)^2^ + 1.8036*P*] where *P* = (*F*_o_^2^ + 2*F*_c_^2^)/3
4979 reflections	(Δ/σ)_max_ < 0.001
403 parameters	Δρ_max_ = 0.53 e Å^−^^3^
6 restraints	Δρ_min_ = −0.52 e Å^−^^3^

### Special details

**Table:**

Geometry. All s.u.'s (except the s.u. in the dihedral angle between two l.s. planes) are estimated using the full covariance matrix. The cell s.u.'s are taken into account individually in the estimation of s.u.'s in distances, angles and torsion angles; correlations between s.u.'s in cell parameters are only used when they are defined by crystal symmetry. An approximate (isotropic) treatment of cell s.u.'s is used for estimating s.u.'s involving l.s. planes.
Refinement. Refinement of *F*^2^ against ALL reflections. The weighted *R*-factor *wR* and goodness of fit *S* are based on *F*^2^, conventional *R*-factors *R* are based on *F*, with *F* set to zero for negative *F*^2^. The threshold expression of *F*^2^ > σ(*F*^2^) is used only for calculating *R*-factors(gt) *etc*. and is not relevant to the choice of reflections for refinement. *R*-factors based on *F*^2^ are statistically about twice as large as those based on *F*, and *R*- factors based on ALL data will be even larger.

### Fractional atomic coordinates and isotropic or equivalent isotropic displacement parameters (Å^2^)

**Table:**

	*x*	*y*	*z*	*U*_iso_*/*U*_eq_	
Cu1	0.48017 (5)	0.82083 (4)	0.17536 (4)	0.03778 (16)	
Cu2	0.69669 (5)	0.67765 (4)	0.40785 (4)	0.03492 (16)	
Cl1	0.71789 (11)	0.96716 (9)	0.47942 (8)	0.0404 (2)	
Cl2	0.18182 (13)	0.66397 (10)	−0.12121 (9)	0.0502 (3)	
O1	0.4879 (3)	0.6029 (3)	0.3599 (2)	0.0397 (6)	
O2	0.5561 (3)	0.7179 (3)	0.0997 (2)	0.0421 (7)	
O3P	0.8668 (4)	1.0066 (3)	0.5557 (3)	0.0625 (9)	
O4P	0.6998 (4)	1.0681 (3)	0.4620 (3)	0.0635 (9)	
O5P	0.6818 (4)	0.8682 (3)	0.3721 (3)	0.0620 (9)	
O6P	0.6191 (5)	0.9214 (4)	0.5281 (4)	0.0790 (12)	
O7P	0.2836 (5)	0.7790 (4)	−0.0273 (3)	0.0841 (13)	
O8P	0.1176 (7)	0.5817 (4)	−0.0787 (5)	0.1186 (19)	
O9P	0.2632 (8)	0.6193 (5)	−0.1779 (5)	0.129 (2)	
O10P	0.0789 (7)	0.6826 (7)	−0.1963 (6)	0.158 (3)	
N1	0.9185 (4)	0.7628 (3)	0.4620 (3)	0.0377 (7)	
N2	0.6939 (4)	0.6190 (3)	0.2490 (3)	0.0390 (8)	
N3	0.7429 (4)	0.7226 (3)	0.5692 (3)	0.0377 (7)	
N4	0.3318 (4)	0.6842 (3)	0.1875 (3)	0.0418 (8)	
N5	0.3875 (4)	0.9249 (3)	0.2452 (3)	0.0435 (8)	
N6	0.5927 (4)	0.9644 (3)	0.1598 (3)	0.0438 (8)	
C1	0.8468 (5)	0.6837 (4)	0.2588 (4)	0.0447 (10)	
H1A	0.8627	0.7639	0.2594	0.054*	
H1B	0.8605	0.6354	0.1936	0.054*	
C2	0.9551 (5)	0.6995 (4)	0.3685 (4)	0.0463 (10)	
H2A	0.9466	0.6195	0.3651	0.056*	
H2B	1.0558	0.7486	0.3811	0.056*	
C3	0.9887 (5)	0.7633 (5)	0.5735 (4)	0.0487 (11)	
H3A	1.0910	0.8255	0.6129	0.058*	
H3B	0.9879	0.6832	0.5618	0.058*	
C4	0.9022 (5)	0.7919 (5)	0.6450 (4)	0.0490 (11)	
H4A	0.9244	0.7675	0.7071	0.059*	
H4B	0.9293	0.8800	0.6782	0.059*	
C5	0.6476 (5)	0.6967 (4)	0.6119 (3)	0.0384 (9)	
H5A	0.6836	0.7164	0.6900	0.046*	
C6	0.4894 (4)	0.6394 (4)	0.5491 (3)	0.0354 (8)	
C7	0.4020 (5)	0.6276 (4)	0.6131 (4)	0.0468 (10)	
H7	0.4484	0.6527	0.6917	0.056*	
C8	0.2508 (5)	0.5801 (4)	0.5621 (4)	0.0499 (11)	
H8	0.1954	0.5762	0.6062	0.060*	
C9	0.1807 (5)	0.5377 (4)	0.4439 (4)	0.0475 (11)	
H9	0.0778	0.5045	0.4086	0.057*	
C10	0.2621 (4)	0.5444 (4)	0.3793 (4)	0.0395 (9)	
H10	0.2125	0.5132	0.3000	0.047*	
C11	0.4171 (4)	0.5967 (3)	0.4277 (4)	0.0356 (8)	
C12	0.2444 (5)	0.7334 (5)	0.2428 (4)	0.0508 (11)	
H12A	0.2914	0.7627	0.3254	0.061*	
H12B	0.1454	0.6686	0.2147	0.061*	
C13	0.2352 (5)	0.8390 (4)	0.2150 (4)	0.0468 (10)	
H13A	0.1754	0.8082	0.1338	0.056*	
H13B	0.1905	0.8804	0.2591	0.056*	
C14	0.4064 (6)	1.0212 (4)	0.2021 (4)	0.0516 (11)	
H14A	0.3897	1.0875	0.2487	0.062*	
H14B	0.3349	0.9858	0.1233	0.062*	
C15	0.5611 (6)	1.0710 (4)	0.2080 (4)	0.0539 (12)	
H15A	0.5690	1.1190	0.1639	0.065*	
H15B	0.6315	1.1243	0.2871	0.065*	
C16	0.6800 (5)	0.9681 (4)	0.1085 (4)	0.0508 (11)	
H16	0.7260	1.0420	0.1046	0.061*	
C17	0.7135 (5)	0.8673 (4)	0.0559 (4)	0.0458 (10)	
C18	0.8147 (6)	0.8893 (5)	0.0056 (5)	0.0629 (14)	
H18	0.8590	0.9681	0.0102	0.076*	
C19	0.8513 (7)	0.8000 (6)	−0.0501 (5)	0.0713 (16)	
H19	0.9198	0.8177	−0.0823	0.086*	
C20	0.7843 (7)	0.6823 (6)	−0.0578 (5)	0.0708 (15)	
H20	0.8079	0.6202	−0.0953	0.085*	
C21	0.6831 (6)	0.6569 (5)	−0.0100 (4)	0.0576 (12)	
H21	0.6374	0.5768	−0.0180	0.069*	
C22	0.6463 (5)	0.7482 (4)	0.0505 (3)	0.0425 (10)	
H1	0.939 (5)	0.836 (2)	0.468 (4)	0.037 (11)*	
H2	0.670 (5)	0.542 (2)	0.220 (4)	0.051 (14)*	
H3	0.636 (4)	0.635 (4)	0.203 (3)	0.035 (11)*	
H4	0.275 (4)	0.629 (3)	0.1181 (19)	0.025 (10)*	
H5	0.370 (4)	0.651 (4)	0.227 (3)	0.039 (12)*	
H6	0.435 (4)	0.960 (4)	0.3166 (18)	0.040 (12)*	

### Atomic displacement parameters (Å^2^)

**Table:**

	*U*^11^	*U*^22^	*U*^33^	*U*^12^	*U*^13^	*U*^23^
Cu1	0.0470 (3)	0.0347 (3)	0.0394 (3)	0.0218 (2)	0.0218 (2)	0.0175 (2)
Cu2	0.0324 (3)	0.0383 (3)	0.0361 (3)	0.0163 (2)	0.0152 (2)	0.0164 (2)
Cl1	0.0419 (5)	0.0336 (5)	0.0399 (5)	0.0184 (4)	0.0122 (4)	0.0107 (4)
Cl2	0.0618 (7)	0.0415 (6)	0.0332 (5)	0.0193 (5)	0.0111 (5)	0.0091 (4)
O1	0.0364 (15)	0.0482 (16)	0.0372 (14)	0.0171 (13)	0.0167 (12)	0.0215 (13)
O2	0.0559 (17)	0.0396 (15)	0.0442 (16)	0.0262 (14)	0.0294 (14)	0.0210 (13)
O3P	0.0487 (19)	0.0512 (19)	0.065 (2)	0.0210 (16)	0.0049 (16)	0.0148 (16)
O4P	0.062 (2)	0.0436 (18)	0.069 (2)	0.0254 (16)	0.0070 (17)	0.0193 (16)
O5P	0.081 (2)	0.054 (2)	0.0428 (17)	0.0379 (18)	0.0181 (17)	0.0074 (15)
O6P	0.089 (3)	0.073 (2)	0.091 (3)	0.032 (2)	0.063 (2)	0.035 (2)
O7P	0.111 (3)	0.057 (2)	0.045 (2)	0.019 (2)	0.012 (2)	0.0098 (17)
O8P	0.147 (5)	0.072 (3)	0.105 (4)	0.005 (3)	0.058 (4)	0.037 (3)
O9P	0.226 (7)	0.106 (4)	0.137 (5)	0.110 (4)	0.134 (5)	0.063 (4)
O10P	0.103 (4)	0.177 (6)	0.149 (6)	0.051 (4)	−0.024 (4)	0.089 (5)
N1	0.0353 (18)	0.0349 (19)	0.0467 (19)	0.0161 (15)	0.0168 (15)	0.0212 (16)
N2	0.043 (2)	0.0359 (19)	0.0425 (19)	0.0210 (16)	0.0195 (16)	0.0169 (16)
N3	0.0327 (17)	0.0438 (19)	0.0343 (17)	0.0160 (15)	0.0100 (14)	0.0178 (15)
N4	0.046 (2)	0.042 (2)	0.042 (2)	0.0199 (17)	0.0227 (17)	0.0184 (17)
N5	0.052 (2)	0.044 (2)	0.0327 (18)	0.0267 (17)	0.0125 (16)	0.0124 (16)
N6	0.051 (2)	0.0343 (18)	0.0431 (19)	0.0203 (16)	0.0135 (17)	0.0169 (16)
C1	0.049 (2)	0.043 (2)	0.051 (2)	0.023 (2)	0.028 (2)	0.021 (2)
C2	0.035 (2)	0.044 (2)	0.062 (3)	0.0191 (19)	0.023 (2)	0.020 (2)
C3	0.034 (2)	0.056 (3)	0.057 (3)	0.020 (2)	0.014 (2)	0.029 (2)
C4	0.042 (2)	0.058 (3)	0.041 (2)	0.019 (2)	0.0086 (19)	0.024 (2)
C5	0.045 (2)	0.036 (2)	0.036 (2)	0.0192 (18)	0.0165 (18)	0.0162 (17)
C6	0.042 (2)	0.034 (2)	0.042 (2)	0.0214 (17)	0.0226 (18)	0.0206 (17)
C7	0.066 (3)	0.036 (2)	0.052 (3)	0.027 (2)	0.037 (2)	0.021 (2)
C8	0.052 (3)	0.052 (3)	0.070 (3)	0.030 (2)	0.040 (2)	0.035 (2)
C9	0.041 (2)	0.046 (2)	0.074 (3)	0.024 (2)	0.032 (2)	0.036 (2)
C10	0.035 (2)	0.041 (2)	0.049 (2)	0.0165 (18)	0.0154 (18)	0.0275 (19)
C11	0.039 (2)	0.032 (2)	0.046 (2)	0.0195 (17)	0.0226 (18)	0.0204 (17)
C12	0.053 (3)	0.058 (3)	0.051 (3)	0.029 (2)	0.030 (2)	0.022 (2)
C13	0.047 (2)	0.053 (3)	0.042 (2)	0.029 (2)	0.019 (2)	0.015 (2)
C14	0.069 (3)	0.048 (3)	0.047 (2)	0.037 (2)	0.021 (2)	0.021 (2)
C15	0.068 (3)	0.040 (2)	0.053 (3)	0.026 (2)	0.018 (2)	0.021 (2)
C16	0.055 (3)	0.047 (3)	0.048 (2)	0.014 (2)	0.017 (2)	0.028 (2)
C17	0.045 (2)	0.052 (3)	0.043 (2)	0.020 (2)	0.021 (2)	0.024 (2)
C18	0.060 (3)	0.070 (3)	0.067 (3)	0.021 (3)	0.032 (3)	0.041 (3)
C19	0.071 (4)	0.096 (4)	0.063 (3)	0.036 (3)	0.050 (3)	0.036 (3)
C20	0.076 (4)	0.079 (4)	0.058 (3)	0.034 (3)	0.039 (3)	0.019 (3)
C21	0.071 (3)	0.054 (3)	0.054 (3)	0.028 (2)	0.037 (3)	0.018 (2)
C22	0.047 (2)	0.047 (2)	0.034 (2)	0.022 (2)	0.0151 (18)	0.0166 (18)

### Geometric parameters (Å, °)

**Table:**

Cu1—O2	1.891 (3)	C3—C4	1.516 (6)
Cu1—N6	1.933 (4)	C3—H3A	0.9700
Cu1—N5	2.005 (4)	C3—H3B	0.9700
Cu1—N4	2.006 (4)	C4—H4A	0.9700
Cu2—O1	1.895 (3)	C4—H4B	0.9700
Cu2—N3	1.930 (3)	C5—C6	1.436 (6)
Cu2—N2	2.010 (4)	C5—H5A	0.9300
Cu2—N1	2.012 (3)	C6—C7	1.410 (6)
Cl1—O4P	1.422 (3)	C6—C11	1.429 (6)
Cl1—O3P	1.425 (3)	C7—C8	1.367 (7)
Cl1—O6P	1.426 (4)	C7—H7	0.9300
Cl1—O5P	1.435 (3)	C8—C9	1.390 (7)
Cl2—O10P	1.391 (5)	C8—H8	0.9300
Cl2—O9P	1.406 (5)	C9—C10	1.364 (6)
Cl2—O8P	1.410 (5)	C9—H9	0.9300
Cl2—O7P	1.411 (4)	C10—C11	1.398 (6)
O1—C11	1.317 (5)	C10—H10	0.9300
O2—C22	1.311 (5)	C12—C13	1.514 (6)
N1—C3	1.472 (6)	C12—H12A	0.9700
N1—C2	1.472 (5)	C12—H12B	0.9700
N1—H1	0.851 (19)	C13—H13A	0.9700
N2—C1	1.487 (5)	C13—H13B	0.9700
N2—H2	0.849 (19)	C14—C15	1.499 (7)
N2—H3	0.859 (19)	C14—H14A	0.9700
N3—C5	1.276 (5)	C14—H14B	0.9700
N3—C4	1.472 (5)	C15—H15A	0.9700
N4—C12	1.476 (5)	C15—H15B	0.9700
N4—H4	0.861 (19)	C16—C17	1.442 (7)
N4—H5	0.860 (19)	C16—H16	0.9300
N5—C13	1.466 (6)	C17—C18	1.396 (7)
N5—C14	1.483 (6)	C17—C22	1.414 (6)
N5—H6	0.836 (19)	C18—C19	1.365 (8)
N6—C16	1.273 (6)	C18—H18	0.9300
N6—C15	1.476 (6)	C19—C20	1.386 (8)
C1—C2	1.500 (6)	C19—H19	0.9300
C1—H1A	0.9700	C20—C21	1.375 (7)
C1—H1B	0.9700	C20—H20	0.9300
C2—H2A	0.9700	C21—C22	1.408 (6)
C2—H2B	0.9700	C21—H21	0.9300
			
O2—Cu1—N6	95.35 (14)	H3A—C3—H3B	108.4
O2—Cu1—N5	176.01 (13)	N3—C4—C3	108.1 (4)
N6—Cu1—N5	85.05 (16)	N3—C4—H4A	110.1
O2—Cu1—N4	94.12 (14)	C3—C4—H4A	110.1
N6—Cu1—N4	168.50 (15)	N3—C4—H4B	110.1
N5—Cu1—N4	85.06 (15)	C3—C4—H4B	110.1
O1—Cu2—N3	95.44 (13)	H4A—C4—H4B	108.4
O1—Cu2—N2	96.03 (13)	N3—C5—C6	125.3 (4)
N3—Cu2—N2	164.80 (14)	N3—C5—H5A	117.3
O1—Cu2—N1	177.95 (13)	C6—C5—H5A	117.3
N3—Cu2—N1	83.63 (14)	C7—C6—C11	118.9 (4)
N2—Cu2—N1	85.21 (14)	C7—C6—C5	117.1 (4)
O4P—Cl1—O3P	110.2 (2)	C11—C6—C5	124.0 (4)
O4P—Cl1—O6P	108.8 (3)	C8—C7—C6	121.5 (4)
O3P—Cl1—O6P	109.3 (3)	C8—C7—H7	119.2
O4P—Cl1—O5P	110.8 (2)	C6—C7—H7	119.2
O3P—Cl1—O5P	109.6 (2)	C7—C8—C9	119.4 (4)
O6P—Cl1—O5P	108.0 (2)	C7—C8—H8	120.3
O10P—Cl2—O9P	108.8 (4)	C9—C8—H8	120.3
O10P—Cl2—O8P	113.7 (4)	C10—C9—C8	120.3 (4)
O9P—Cl2—O8P	109.6 (4)	C10—C9—H9	119.8
O10P—Cl2—O7P	108.9 (4)	C8—C9—H9	119.8
O9P—Cl2—O7P	107.0 (4)	C9—C10—C11	122.5 (4)
O8P—Cl2—O7P	108.7 (3)	C9—C10—H10	118.8
C11—O1—Cu2	126.0 (3)	C11—C10—H10	118.8
C22—O2—Cu1	125.5 (3)	O1—C11—C10	118.9 (4)
C3—N1—C2	116.0 (3)	O1—C11—C6	123.9 (3)
C3—N1—Cu2	108.7 (3)	C10—C11—C6	117.2 (4)
C2—N1—Cu2	106.7 (3)	N4—C12—C13	108.9 (4)
C3—N1—H1	111 (3)	N4—C12—H12A	109.9
C2—N1—H1	107 (3)	C13—C12—H12A	109.9
Cu2—N1—H1	106 (3)	N4—C12—H12B	109.9
C1—N2—Cu2	108.0 (3)	C13—C12—H12B	109.9
C1—N2—H2	107 (3)	H12A—C12—H12B	108.3
Cu2—N2—H2	111 (3)	N5—C13—C12	106.9 (4)
C1—N2—H3	108 (3)	N5—C13—H13A	110.3
Cu2—N2—H3	113 (3)	C12—C13—H13A	110.3
H2—N2—H3	110 (4)	N5—C13—H13B	110.3
C5—N3—C4	119.9 (3)	C12—C13—H13B	110.3
C5—N3—Cu2	125.1 (3)	H13A—C13—H13B	108.6
C4—N3—Cu2	115.0 (3)	N5—C14—C15	108.8 (4)
C12—N4—Cu1	109.1 (3)	N5—C14—H14A	109.9
C12—N4—H4	110 (3)	C15—C14—H14A	109.9
Cu1—N4—H4	106 (3)	N5—C14—H14B	109.9
C12—N4—H5	108 (3)	C15—C14—H14B	109.9
Cu1—N4—H5	114 (3)	H14A—C14—H14B	108.3
H4—N4—H5	110 (4)	N6—C15—C14	107.6 (4)
C13—N5—C14	117.2 (4)	N6—C15—H15A	110.2
C13—N5—Cu1	106.1 (3)	C14—C15—H15A	110.2
C14—N5—Cu1	106.3 (3)	N6—C15—H15B	110.2
C13—N5—H6	110 (3)	C14—C15—H15B	110.2
C14—N5—H6	109 (3)	H15A—C15—H15B	108.5
Cu1—N5—H6	108 (3)	N6—C16—C17	125.4 (4)
C16—N6—C15	121.7 (4)	N6—C16—H16	117.3
C16—N6—Cu1	125.1 (3)	C17—C16—H16	117.3
C15—N6—Cu1	113.0 (3)	C18—C17—C22	119.3 (4)
N2—C1—C2	108.4 (3)	C18—C17—C16	117.3 (4)
N2—C1—H1A	110.0	C22—C17—C16	123.4 (4)
C2—C1—H1A	110.0	C19—C18—C17	122.7 (5)
N2—C1—H1B	110.0	C19—C18—H18	118.7
C2—C1—H1B	110.0	C17—C18—H18	118.7
H1A—C1—H1B	108.4	C18—C19—C20	118.6 (5)
N1—C2—C1	106.9 (3)	C18—C19—H19	120.7
N1—C2—H2A	110.3	C20—C19—H19	120.7
C1—C2—H2A	110.3	C21—C20—C19	120.3 (5)
N1—C2—H2B	110.3	C21—C20—H20	119.9
C1—C2—H2B	110.3	C19—C20—H20	119.9
H2A—C2—H2B	108.6	C20—C21—C22	122.3 (5)
N1—C3—C4	108.3 (3)	C20—C21—H21	118.9
N1—C3—H3A	110.0	C22—C21—H21	118.9
C4—C3—H3A	110.0	O2—C22—C21	118.2 (4)
N1—C3—H3B	110.0	O2—C22—C17	125.0 (4)
C4—C3—H3B	110.0	C21—C22—C17	116.8 (4)

### Hydrogen-bond geometry (Å, °)

**Table:**

*D*—H···*A*	*D*—H	H···*A*	*D*···*A*	*D*—H···*A*
N1—H1···O3P^i^	0.85 (2)	2.52 (3)	3.188 (5)	136 (4)
N1—H1···O3P	0.85 (2)	2.54 (3)	3.208 (5)	136 (4)
N2—H2···O9P^ii^	0.85 (2)	2.30 (3)	3.069 (6)	151 (4)
N2—H3···O2	0.86 (2)	2.12 (2)	2.955 (5)	162 (4)
N4—H4···O8P	0.86 (2)	2.46 (3)	3.223 (7)	148 (3)
N4—H5···O1	0.86 (2)	2.24 (2)	3.084 (5)	168 (4)
N5—H6···O6P^iii^	0.84 (2)	2.47 (4)	3.081 (5)	131 (4)
N5—H6···O4P	0.84 (2)	2.51 (3)	3.195 (5)	139 (4)

Symmetry codes: (i) −*x*+2, −*y*+2, −*z*+1; (ii) −*x*+1, −*y*+1, −*z*; (iii) −*x*+1, −*y*+2, −*z*+1.
